# Provision of Care to the People with HIV: Voices of Professional Nurses in the Public Hospitals of Limpopo Province, South Africa

**DOI:** 10.3390/ijerph18063112

**Published:** 2021-03-18

**Authors:** Maria Lebeko Moshidi, Rambelani Nancy Malema, Livhuwani Muthelo, Tebogo Maria Mothiba

**Affiliations:** 1 Department of Nursing Science, University of Limpopo, Sovenga 0727, South Africa; mariamoshidi@gmail.com (M.L.M.); nancy.malema@ul.ac.za (R.N.M.); 2 Faculty of Health Sciences Executive Dean’s Office, University of Limpopo, Sovenga 0727, South Africa; tebogo.mothiba@ul.ac.za

**Keywords:** HIV and AIDS, professional nurses, public hospital and care

## Abstract

The battle against the Human Immunodeficiency Virus (HIV) and Acquired Immunodeficiency Syndrome (AIDS) epidemic are still raging in South Africa (SA) despite all the preventive strategies implemented via the five-year strategic plan (2011–2015). The intensity of the AIDS pandemic in SA creates additional challenges for the health workers as they have to deal with an increasing number of people who suffer from this disease. Professional nurses are a critical part of the workforce. The qualitative, explorative, descriptive, and contextual study design was conducted in five public hospitals from each district of Limpopo Province. The purpose of the study was to explore and describe experiences regarding support received by professional nurses while providing care to HIV and AIDS patients in the public hospitals of Limpopo Province. Purposive sampling was employed to select the participants who provided care to HIV and AIDS patients for 24 months or more. The recruitment of participants continued until data saturation was reached at participant number 20. Data were collected through face-to-face individual interviews using a semi-structured guide. Data were analyzed using Tech’s qualitative data analysis method. Trustworthiness was measured through credibility, dependability, confirmability, and transferability. Findings: Emotional and physical strain due to a shortage of staff, heavy workload, staff turnover, and high absenteeism were experienced by the nurses fulfilling these tasks. Exhaustion, fatigue, increased levels of stress, and lack of training, counselling, debriefing sessions, recognitions, and reward systems were also experienced. Recommendations: A program for support of all professional nurses providing care to HIV and AIDS patients should be conceptualized and implemented.

## 1. Introduction 

The incidence of HIV infection has stabilized globally and begun to decline in many countries with a generalized epidemic. According to 2020 Global HIV and AIDS statistics, 38.0 million people globally were living with HIV in 2019 with 1.7 million new infections and 26.0 million accessing antiretroviral therapy at the end of June 2020. Furthermore, 690,000 people died from AIDS in 2019 [1]. There was a decline from 2.1 million new infections in 2015 to 1.8 million new HIV infections in 2016 [2]. Despite these advances, still too many people are contracting HIV infections and too many people are becoming sick and dying. Bell indicated that an estimated 31% of children in the United States have one or more chronic illnesses that often require frequent hospitalization [3]. A study conducted in New York City by Grant M et al. found that hospital health care workers were capable of handling many of the palliative care needs of patients, but these patients often died [4]. 

Sub-Saharan Africa remains the most severely affected, making it the world’s epicentre of HIV and AIDS, although it contains only about 11% of the Earth’ population [1]. The HIV prevalence in adults is 1.2% worldwide, with 9.0% of the people living in Sub Saharan Africa. In 2018, SA has the biggest HIV epidemic in the world with 7.7 million people living with HIV [1]. The highest rate of new infections in SA has been identified amongst adolescent girls and young women [5,6]. According to Mswela M, various factors arising from cultural practices increase women’s vulnerability to HIV in SA [7]. Such factors include poverty, the low regard for women, and gender-based violence against black women, especially in rural areas [7]. The current study was conducted in Limpopo Province, which is one of the rural provinces in SA, where hospitals are congested with HIV patients. According to Statistics South Africa, the prevalence of HIV in Limpopo Province was 17% in 2017 with 259,227 new HIV infections. Additionally, the burden of HIV in Limpopo Province affects hospital admissions, with 13% of patients being admitted due to HIV-related conditions [8]. These patients are hospitalised, suffering from fatal opportunistic infections and secondary complications due to a depleted immune system. The National Strategic Plan 2012–2016 aims to lessen the impact of HIV and AIDS on people. In the high demand for effectiveness and efficiency of public health service delivery, the nursing staff is given a huge responsibility to ensure that the demands of the society are satisfied [9]. The increase in the admission rate of HIV patients who are terminally ill in public hospitals contributes to exhaustion, stress, and burnout among professional nurses [10]. The impact of caring for patients who are terminally ill as well as their families may prove to be overwhelming for the professional nurses if support systems, particularly in the work environment, are not put in place [11].

Throughout the world, HIV is one of the main health challenges which impact human resources for adequate patient care [2] The study conducted in KwaZulu-Natal, SA by Singh D et al. revealed that caregivers of patients with HIV and AIDS were experiencing high levels of emotional and physical burden due to the demands of their jobs [12]. The increasing burden of HIV in SA and care and support for HIV and AIDS patients has brought a great need for people who care for these patients [13]. The expansion of treatment for HIV posed more stress to caregivers, as more people living with HIV and AIDS had prolonged life expectancy due to antiretroviral treatment (ART) [13]. According to the Joint United Nations Programme on HIVand AIDS (UNAIDS), the decline in deaths from AIDS-related illnesses was sharpest in Eastern and Southern Africa where they peaked at 1.1 million in 2004 and dropped by 62% to 420,000 in 2016 due to the rapid up-scale of antiretrovirals (ARVs) in the region [2].

Providing nursing care to HIV and AIDS patients means dealing with the patients as well as their affected families. Nurses are on the front line providing both physical and emotional support for patients and their families [14]. The study conducted by Valjee C and van Dyk AC reported that providing care for individuals with serious chronic illnesses is a physical and emotional challenge that has a detrimental effect on the well-being of caregivers if not effectively managed [15]. Professional nurses were generally experiencing emotional and physical strain when providing care to terminally ill AIDS patients. This prompted the researcher to explore and describe the experiences of professional nurses providing care to HIV and AIDS patients in the public hospitals of Limpopo Province. 

## 2. Methodology

### 2.1. Study Design and Setting

A qualitative, explorative, descriptive, and contextual research design was utilized to obtain complete and accurate information about the experiences of professional nurses regarding the support they received, while they are providing care to HIV and AIDS patients in the public hospitals of Limpopo Province. The study was conducted at five selected public hospitals, one from each district of Limpopo Province. These hospitals were selected because they all had similar characteristics of having HIV and AIDS patients. Limpopo Province has five districts and 40 hospitals. The regional hospitals were selected because they are the referral hospitals for the district hospitals. The Capricorn district does not have a regional hospital, so one hospital was selected since most HIV and AIDS patients are admitted there, as compared to the other district hospitals. 

### 2.2. Population and Sampling 

The research population for this study comprised professional nurses from five public hospitals in Limpopo Province, who had provided care to HIV and AIDS patients in the medical wards and have been working in those wards for twenty-four months or more. A purposive sampling method was used to select two professional nurses from each ward, meaning that four professional nurses per regional hospital were selected. The sample size was determined by data saturation being reached after 20 interviews from the respectively selected five public hospitals.

### 2.3. Data Collection Procedure

Data were collected through in-depth interviews using the semi-structured interview guide, written in English, a language that was understandable to all participants. Participants were asked the same questions, in the same order, to maintain consistency, followed by probing questions until data saturation was reached. The central question that was posed was: “What are the experiences of professional nurses regarding the support received while providing care to HIV/AIDS patients in the public hospitals of Limpopo Province”. Individuals’ verbal information was captured on a voice recorder. Field notes were also taken to capture non-verbal cues. 

### 2.4. Data Analysis

Data were analyzed using an open-coding method following Tech’s qualitative data analysis method [16]. Data within the same category were then grouped and subcategories developed. Meaningful units were identified and coded. Common and unique themes and sub-themes were identified [17]. A co-coder was engaged to analyze the data independently. The researcher and the co-coder reached a consensus regarding the identified themes and sub-themes. 

### 2.5. Trustworthiness 

Trustworthiness was maintained following four criteria of the Lincoln and Guba model (De Vos AS et al. namely: Credibility (truth value), confirmability (neutrality), dependability (consistency), and transferability (applicability) [18]. Credibility and confirmability were ensured through triangulation and prolonged engagement with participants. Triangulation was enhanced through the use of in-depth interviews, observations, and field notes to develop a comprehensive understanding of the phenomenon being studied. Prolonged engagement with the participants, for about three weeks, was ensured, until data saturation was reached. The copies of the verbatim transcripts, together with the field notes that were taken, were sent to the independent coder for analysis. Identified themes and sub-themes were compared with those of the researcher.

Dependability and transferability were ensured through the use of a detailed description of the methodology used that described the experiences regarding support received by professional nurses while providing care to HIV and AIDS patients in the public hospitals of Limpopo Province to assist the researchers who may want to replicate the study. 

### 2.6. Ethical Considerations

To ensure ethical conduct the researcher utilized aspects from Polit DF and Beck CT, which included beneficence, fairness, and respect for others [17]. The ethical clearance was obtained from the Turfloop Research Ethics Committee (TREC/211/2015: PG). Approval to conduct the study was granted by the Department of Health, Limpopo Province. The written permission to conduct the study was sought from the selected hospitals’ authorities. Voluntary informed written consent from participants was secured before data collection. Anonymity and confidentiality were ensured throughout the study. The participants’ right to fair selection and privacy was enforced. The principles of beneficence, which involve doing good, preventing, and removing potential harms, were also confirmed. The research question asked was the same for all the participants. Participants were all given adequate time to express themselves fully and made use of English, a language understandable to all. 

## 3. Results

The findings are presented in a narrative format with participants’ direct quotations written in italics. The purpose of this study was to explore the experiences of professional nurses providing care to HIV and AIDS patients in the public hospitals of Limpopo Province.

### 3.1. Participants’ Demographic Data 

Twenty professional nurses from five public hospitals in five districts of Limpopo Province participated in the semi-structured face-to-face interviews. Four professional nurses were drawn from each public hospital of Limpopo Province, namely: Mokopane Hospital (Waterberg district), Tshilidzini Hospital (Vhembe district), Philadelphia Hospital (Sekhukhune district), Seshego Hospital (Capricorn district), and Letaba Hospital (Mopane district). All participants had a basic professional nursing diploma. The average years of experience in medical departments were 10 years and ranged from 3 to 20 years. The ages were between 29 and 50 years. Educational qualifications were as follows: One professional nurse was trained in oncology, one was trained on palliative care, two had a diploma in psychiatric nursing and four were trained comprehensively (R425), and three had general nursing (R683) only and were to commence midwifery training (R254) in June 2016. Eight professional nurses had both a diploma in general nursing and midwifery. All professional nurses worked in medical wards. 

### 3.2. Themes and Sub-Themes

The findings of the study were organized into themes and sub-themes that emerged during data analysis. Themes and sub-themes enforced by direct quotes from participants are presented in Table 1: The direct quotes from participants are presented in italics.

**Theme** **1.** *Explanation of accounts related to experiences during the provision of care and support of HIV and AIDS patients.*

Theme 1 outlines the description given by participants regarding their experiences during the provision of care and support of HIV and AIDS patients. Under this theme, the following two sub-themes emerged, namely: Exhaustion and fatigue experienced during the provision of care and lack of adherence to treatment by patients.

**Sub-theme** **1.1.** *Exhaustion and fatigue experienced during the provision of care.*

Increased workload and patient dependency appeared to be the major challenges expressed by participants, which resulted in physical and psychological exhaustion experienced by the professional nurses. The following verbatim statements mentioned below by participants reflect the extent of exhaustion and fatigue these professional nurses experienced.

**Participant** **03** **from** **public** **hospital** **B:** *“Most of the time we find that the ward is overflowing having 35 to 40 patients with two professional nurses, one staff nurse and one or two assistant nurses. This puts more strain on us as most of the patients are dependent on us, as a result, we sometimes are not able to cope with the workload due to fatigue.”*

**Participant** **01** **from** **public** **hospital** **A:** *“Sometimes you don’t know what tea time is because you sometimes find that almost everybody is being bed bathed, followed by doctors’ ward rounds who need to be accompanied during rounds. That is a real challenge and a stressful situation.”*

**Participant** **04** **from** **public** **hospital** **D:** *“Most of the patients are bed-ridden. They need to be bathed, fed, changed position, given treatment, and we have to do additional jobs of auditing patients’ files and assisting doctors during rounds. You can’t even go for lunch because you find that the ward will be left with staff nurses or nursing assistants only.”*

**Participant** **02** **from** **public** **hospital** **C:** *“Mind you, the medical ward is hectic because most of our patients are HIV-positive and are bedridden, they can’t help themselves.”*

**Sub-theme** **1.2.** *Lack of adherence to treatment by patients.*

Keeping patients on treatment programs is imperative and the rise in patients failing to follow up their ART is particularly worrying. Participants expressed their challenges in the following verbal statements:

**Participant** **03** **public** **hospital** **D:** *“Most of the patients with HIV/AIDS don’t comply with their treatment and this poses more strain on us as workers.”*

**Participant** **03** **public** **hospital** **B:** *“Most patients don’t comply with their ARV treatment. Some collect their treatment from traditional healers. When they come back to the hospital you will find that their CD4 count will be low and their viral load high.”*

**Participant** **03** **from** **public** **hospital** **C:** *“Some of the patients when they are discharged they don’t take their treatment at home. They default their treatment and then come back being critically ill.”*

**Participant** **02** **from** **public** **hospital** **A:** *“When patients are discharged with the ARV treatment some of them don’t collect their treatment when finished but decide to go to the traditional healers or the pastors. When they come back you find that those patients’ conditions have deteriorated, being unable to help themselves.”*

**Theme** **2.** *Challenges experienced during provision of care and support of HIV and AIDS patients.*

Caregiving of HIV and AIDS patients has placed considerable demands on the professional nurses providing care to the infected patients, which are exacerbated by insufficient support. In the high demand for effectiveness and efficiency of public health service delivery, the nursing staff is burdened with great responsibility to ensure that the demands of public citizens are satisfied. Six sub-themes that emerged from theme 2 are supported by the verbatim quotations of participants written in italics.

**Sub-theme** **2.1.** *Lack of management support experienced.*

Support plays an important role in increasing nurses’ self-confidence and leading to clinical competency and productivity [19]. Professional nurses experienced a lack of support in their work because they were not provided with adequate human resources, which resulted in an increased workload. Organizing support groups amongst co-workers was identified by participants as one of the needs management should consider in the workplace as that might add value in boosting their morale and confidence. Participants confirmed the lack of support by saying:

**Participant** **01** **public** **hospital** **E:** *“Maybe if management can organize a support system for us where we can meet with the psychologist to counsel us or meet support groups to share our experiences with them because we see most of the patients dying in our care and this depresses us.”*

**Participant** **01** **public** **hospital** **A:** *“We plea for management presence and support because it is strenuous, the ward is too heavy for us. It seems as if we have been neglected. Let them not only come to pick up the wrong things that we are doing because we are not doing the wrongs always”.*

**Participant** **02** **public** **hospital** **B:** *“So in terms of support, normally there is nothing like support. Management doesn’t come to our ward to even see how we cope even if they can see the high patient statistics whereby we sometimes have close to 35 patients being one or two professional nurses with junior categories.*”

**Sub-theme** **2.2.** *Emotional and psychological trauma experienced during the caring process.*

Physical and psychological trauma experienced by professional nurses were the nature of the work itself. The fact that they were dealing with an incurable condition that killed people, young and adults alike, weigh heavily on the nurses. The caregivers’ burden often produced a high level of chronic stress that needed care and support from the system itself. The following quotes from the professional nurses’ responses indicate these findings.

**Participant** **03** **form** **public** **hospital** **D:** *“Oh! We are so demoralised. You find that people are dying in front of us, some are our families, relatives, and our neighbours. It is traumatizing.”*

**Participant** **02** **from** **public** **hospital** **A:** *“Some of these patients come being terminally ill. Seeing those patients nearly every day suffering from these conditions stresses us, you become affected psychologically and emotionally. There are also no debriefing, no counselling sessions. We need psychological support.”*

**Participant** **02** **from** **public** **hospital** **C:** *“Since I have been working here in the medical ward I never had debriefing sessions nor any counselling session. It is so traumatic and stressful to us and we are sometimes unable to cope properly with the workload. We need support and as I have said managers don’t support us.”*

**Sub-theme** **2.3.** *Shortages of staff being experienced compromises the provision of care and support to patients.*

Although the shortage of nurses remains a global issue, very little is being done to correct this shortage. The following quotes from participants illustrate their responses regarding staff shortages:

**Participant** **04** **from** **public** **hospital** **E:** *“The ward is always full with close to 35 patients and we are short-staffed, we work being two professional nurses in this shift, so it is difficult to run the ward. Sometimes you find that we do ward rounds, followed by administration of medication, admission of patients.”*

**Participant** **02** **from** **public** **hospital** **C:** *“Regarding the issue of shortage of staff, I think management should consider developing a way of recruiting and retaining nurses. The shortage of staff is a real challenge in our ward. We are being short-staffed till to date.’’*

**Participant** **02** **from** **public** **hospital** **E** *“With the shortage of staff that we have it adds more work to us. You start by bathing the patients and mind you, we are only four nurses in the ward, one professional nurse, one staff nurse, and two assistant nurses with 32 patients. All these needs add more work to the skeletal staff that we have.”*

**Sub-theme** **2.4.** *High staff turnover rates and absenteeism experienced.*

Staff turnover is a possible threat to loss of knowledge, whereby knowledgeable and experienced employees happen to leave the organization [20]. Staff turnover and absenteeism remained the challenge experienced in this study. As quoted by the following professional nurses:

**Participant** **02** **from** **public** **hospital** **C:** *“Last year we had two resignations in the medical ward, two of our professional nurses resigned. So apparently some staff members are still planning to leave as far as the situation is concerned.”*

**Participant** **03** **from** **public** **hospital** **A:** *“So at the end of the day things like staff turnover and absenteeism are very high because people are tired. You find it difficult to come to work the following day because of the workload that we are facing. Four professional nurses already resigned in March and this has exacerbated the situation that we are in now.”*

**Participant** **01** **from** **public** **hospital** **C:** *“I remember our junior nurses, staff nurses, and auxiliary nurses, once decided not to come to work because they were tired. They reported that they couldn’t cope with the situation any longer and decided not to come to work the following day.”*

**Sub-theme** **2.5.** *Deprivation of work-related benefits experienced*.

Training is one of the tools for staff development and a retention strategy. Due to the rapid changes taking place in medicine treatment modalities and medical technology, it requires that the nurses and healthcare personnel undergo professional development and education continuously.

**Participant** **04** **public** **hospital** **A:** *“Myself I was never trained for HIV/AIDS or maybe attended any HIV and AIDS counselling or testing workshops. We need to be trained and be knowledgeable about the management of HIV and AIDS patients.”*

**Participant** **01** **from** **public** **hospital** **D:** *“I think it will be better if we can all be trained and know the HIV and AIDS treatment that we are giving as they are changing names now and then and we are not trained to know those names. I think we need to be trained.”*

**Participant** **03** **from** **public** **hospital** **A:** *“I have been working in the medical ward since 2001. You can imagine yourself almost 15 years in this situation without any development.”*

**Participant** **02** **from** **public** **hospital** **C:** *“Coming to the issue of workshops, I was selected to go for a workshop by my operational manager to attend HAART workshop. She approved but at the end of the day, I am being denied the opportunities, but we are expected to provide quality care to the patients.”*

**Sub-theme** **2.6.** *Shortage of material resources leads to poor provision of care and support.*

Findings revealed that inadequate resources were one of the challenges experienced by professional nurses in medical wards. Participants revealed there is a need to have a well-equipped and safe working environment to enhance the provision of adequate quality care to patients. Participants indicated that by saying:

**Participant** **02** **from** **public** **hospital** **E:** *“I am a psychiatric trained nurse, but working in the medical ward, which has mixed patients and we don’t have resources like N95 masks to work with. Sometimes our TB patients complicate and become Multi-Drug Resistance (MDR) patients and this exposes us to an occupational hazard which is an infection in this regard.”*

**Participant** **03** **from** **public** **hospital** **D:** *“We also have the challenge of resources, such as beds, we don’t have enough beds. Some of the beds are broken and with worn-out mattresses that need to be fixed. Patients are congested and this exposes the patients to the risk of infection.”*

**Theme** **3.** *Explanation of support experienced by nurses during the provision of care and support of HIV and AIDS patients.*

Care and support are integral components of the management strategies that health care providers regard as essential for managing HIV and AIDS patients. Participants’ quotes are explained as follows:

**Sub-theme** **3.1.** *Different types of compensation exist.*

The findings revealed that different types of compensation exist, and they caused dissatisfaction. Lack of compensation was identified as one of the challenges experienced by professional nurses providing care to HIV and AIDS patients in medical wards. Managers in the workplace are to cultivate employees’ support through the implementation of different forms of compensation. These were outlined by participants who indicated as follows:

**Participant** **01** **from** **public** **hospital** **A:** *“What the management could do is the giving of incentives like danger allowance as a medical ward is also nursing psychiatric patients who are most of the time violent and dangerous. Most of us professional nurses are not even trained for psychiatry. This will also motivate us.”*

**Participant** **02** **from** **public** **hospital** **C:** *“The appraisal of staff, is really what we are pleading for from the management to try to boost our morale.”*

**Participant** **02** **from** **public** **hospital** **D:** *“We need the incentives that will motivate us, such as Occupation-Specific Dispensation (OSD). Let our managers consider this please, we are pleading.”*

**Sub-theme** **3.2.** *Hope for spiritual support expressed.*

The findings pointed out that the use of prayer at the workplace seems to have a noticeable effect on the health system. The trust these professional nurses had in God gave them the courage that they would go through with whatever situation or circumstances they come across in their wards. These findings are supported by the following direct quotes from the participants:

**Participant** **01** **from** **public** **hospital** **C:** *“You end up praying every day when you come to work for God to give you strength and wisdom like David, then you will manage.”*

**Participant** **02** **from** **public** **hospital** **C:** *“The morning prayers we usually hold gives us a little bit of courage and strength. We counsel each other with the verses from the Bible which reminds us of our commitment to our duties, which in this regard is patient care.”*

**Participant** **03** **from** **public** **hospital** **A:** *“But we are just working. God is with us, indeed God is helping us. Most of the support for us is through the help of God, to tell the truth.”*

**Participant** **03** **from** **public** **hospital** **A:** *“Spiritual counselling is needed because sometimes we are always almost on duty even during Easter holidays or on Sundays, you don’t even have the chance of going to church.”*

## 4. Discussion

The study revealed inadequate support for professional nurses providing care to HIV and AIDS patients in the public hospitals of Limpopo Province. The researchers discovered that participants experienced their environment as non-conducive and stressful to effectively provide quality care to HIV and AIDS patients. Workplace stress has been shown to have a detrimental effect on the health and well-being of employees. Potter P et al. and Booyens SW also outlined that caring for terminally ill patients can generate work-related stress, causing nurses to feel dissatisfied with their employers and mentally exhausted [11,21]. Moreover, participants described their job as emotionally demanding, strenuous, and draining. These findings are also in-line with the results reported by various other researchers who outlined that caring for terminally ill patients is physically, emotionally, and spiritually demanding [22,23,24,25]. Sarode P and Shirsath’s M study findings have shown that the employee’s physical and psychological health can be impacted by the poor working environment, which in turn contributes to job dissatisfaction [26]. A supportive working atmosphere as perceived by the employee is of great significance and may have a direct relationship with how employees engage in their jobs [27]. 

Kwak C et al. further indicated that nurses perform better when they perceive that they are supported by their organizations [28]. Professional nurses must be valued and supported in the work environment for the efforts they put into delivering care to HIV and AIDS patients. Nurse managers are the key persons in this study when it comes to the provision of care and support within the workplace. They need to be approachable and always available for nurses should they need any kind of help. 

Another area of concern from the perspective of professional nurses is the lack of human and material resources. The participants experienced a lot of frustrations and were working under strenuous conditions due to a lack of working equipment. Inadequate equipment and adverse working conditions have been shown to affect employees’ commitment and intention to stay in the organization. There was no way that participants could operate well if staff members were not given adequate supplies to perform at their best. 

Dargahi et al. and Colakoglu U et al. states that employees generally perform better when they are satisfied with their jobs, however, if dissatisfied, intention to withdraw increases [29,30]. Chen SH et al. study have shown that job stress occurred when employees perceive an imbalance between their work demands, capabilities, and resources [31]. Nurses cannot be expected to provide quality service delivery if they do not have the necessary supplies and equipment. Poor working conditions are detrimental to staff morale, motivation, and performance [32]. The improvement of conditions of service for nurses should seriously be considered by hospital management. When there is an unnecessary workload, which is aggravated by a shortage of staff, the result is that the provision of quality care to patients is compromised. Due to staff shortages, some participants manifest their dissatisfaction through decreased performance or increased absenteeism. This, in turn, results in the remaining staff members having difficulty in managing their job responsibilities, which leads to the intention to quit [33]. Supporting this, Ilhami and Cetin have indicated that dissatisfied employees are more likely to quit their job [34]. 

During staff shortages, nurses not only do their job but also do the work of others resulting in stress, dissatisfaction, and subsequently staff turnover [35,36,37]. Chen SH et al. have shown that human resource practitioners’ responsibilities are to identify means of retaining and engaging staff within the system as they are a valuable commodity that is not readily replaced [31]. 

Lack of incentives/performance appraisal was viewed as a challenge experienced by participants. Staff needs to feel that the contributions that they are making to the organization are recognized and that their expertise and experiences are valued [38]. The organization has to maintain its commitment to recognizing and appreciating employees. This will be encouraging and motivating to staff because it addresses their self-esteem, self-actualization, and professional worthiness [39,40]. 

Motivated and committed employees may bring in new and better ways of doing things in an organization [41]. This is also supported by the study conducted by Mohammad AH who stated that motivated employees perform best in the interest of organizational effectiveness [42]. It is, therefore, strongly suggested that the employer investigate the current reward system and recognition, policies, and practices to retain staff. Lack of training and development was expressed by participants as one of the threatening risks for effective provision of quality care for HIV and AIDS patients. Without proper training and development, employees are not keen to take ownership of their job, hence resulting in low production [43]. As a consequence, part of managerial duties is to keep employees focused on their work by developing a conducive work environment to enable staff to perform satisfactorily. Training and developmental opportunities should be available for professionals providing care to HIV and AIDS patients. 

Unless this care and support for the professional nurses are managed effectively, the professional nurses will become demoralized and discouraged, impacting negatively on the provision of quality patient care. As it becomes difficult for the professional nurses to provide quality patient care to an increasing number of HIV and AIDS patients with limited resources, strategies to develop a program for support for all professional nurses providing care to HIV and AIDS patients should be developed which should be conceptualized and implemented. The strategies could assist professional nurses to cope more effectively with the provision of quality patient care through the process of care and support. Therefore, managers need to support and keep employees satisfied at work as this has been shown to lead to a higher level of productivity, less absenteeism, and higher job satisfaction. 

## 5. Recommendations

Policies, guidelines, and programs need to be developed as part of care and support for the caregivers to enable them to continue with effective caregiving. Strategies to curb staff turnover need to be developed. Opportunities for training and continuing professional development should be increased especially for professional nurses providing care to HIV and AIDS patients. Strategies to attract and retain professional nurses in the HIV and AIDS settings should be formulated in the form of incentives such as Occupation Specific Dispensation (OSD), to motivate them. Contributions made by nurses should be recognized and rewarded accordingly based on their different levels, responsibilities, and performance. Stress management should be part of the curriculum for all workshops, seminars, and in-service courses for nurses and nurse managers. Revisiting and re-enforcement of the Employee Assistance Program (EAP) should be done to facilitate employee wellness. Managers should foster an open and honest culture to enable staff members to express their feelings openly or in confidence and learn to deal with their frustration. Since some stressors are arising from the working environment, creating a safe, healthy, and conducive working environment such as avoidance of work overloads, increasing staffing, and providing adequate equipment and supplies are necessary. Regular debriefing sessions through psychologists or professional social workers should be instituted.

A more extensive research study could be undertaken at the same institutions, including all the hospital wards/units and other departments that deal directly with HIV and AIDS patients. This study should further explore and describe the experiences of professional nurses providing care to HIV and AIDS patients to obtain more in-depth information regarding the care and support they receive from a broader perspective, as well as to improve service delivery in health care services. Such an extensive study should be repeated at other hospitals in SA to ensure the improvement of the implementation.

## 6. Limitations of the Study

The study is limited to the experiences of professional nurses providing care to HIV/AIDS patients in the public hospitals of Limpopo Province. The results can therefore not be generalized beyond the study participants.

## 7. Conclusions

It can be concluded, based on the findings of the study, that a collaborative approach between managers, psychologists, and professional nurses is needed to meet the patients’ demand for involvement. It is hoped that management interventions will be instituted to improve service delivery to HIV and AIDS patients by well-supported professional nurses. Recommendations, based on the study, have been made by the researcher to the relevant statutory bodies. It is anticipated that the findings of this study will be beneficial to many health care organizations here and in other countries in their efforts to successfully render care and support, which can improve working conditions in the nursing profession.

## Figures and Tables

**Table 1:** Summary of themes and sub-themes.

Themes	Sub-Themes
1. Explanation of accounts related to experiences during the provision of care and support of HIV and AIDS patients	1.1. Exhaustion and fatigue experienced during the provision of care 1.2. Lack of adherence to treatment by patients
2. Challenges experienced during provision of care and support of HIV and AIDS patients	2.1. Lack of management support experienced 2.2. Emotional and psychological trauma experienced during the caring process 2.3. Shortages of experienced staff compromises the provision of care and support to patients 2.4. High staff turnover rates and absenteeism are experienced 2.5. Deprivation of work-related benefits experienced 2.6. Shortage of material resources leads to poor provision of care and support
3. Explanation of support being experienced by nurses during the provision of care and support of HIV and AIDS patients	3.1. Different types of compensation exist 3.2. Hope for spiritual support expressed

## Data Availability

The data sets used during this study can be made available on request to the readers from the corresponding authors.
